# Analysis of the Impact of Fabric Surface Profiles on the Electrical Conductivity of Woven Fabrics

**DOI:** 10.3390/ma18112456

**Published:** 2025-05-23

**Authors:** Ayalew Gebremariam, Magdalena Tokarska, Nawar Kadi

**Affiliations:** 1 Institute of Architecture of Textiles, Faculty of Material Technologies and Textile Design, Lodz University of Technology, 116 Zeromskiego St., 90-543 Lodz, Poland; magdalena.tokarska@p.lodz.pl; 2 Department of Textile Technology, Faculty of Textiles, Engineering and Business, University of Borås, 501 90 Borås, Sweden; nawar.kadi@hb.se

**Keywords:** electrical anisotropy, surface roughness, surface profile, fabric structure, electrical resistance, wearable electronics, Van der Pauw method

## Abstract

The surface profile and structural alignment of fibers and yarns in fabrics are critical factors affecting the electrical properties of conductive textile surfaces. This study aimed to investigate the impact of fabric surface roughness and the geometrical parameters of woven fabrics on their electrical resistance properties. Surface roughness was assessed using the MicroSpy^®^ Profile profilometer FRT (Fries Research & Technology) Metrology™, while electrical resistance was evaluated using the Van der Pauw method. The findings indicate that rougher fabric surfaces exhibit higher electrical resistance due to surface irregularities and lower yarn compactness. In contrast, smoother fabrics improve conductivity by enhancing surface uniformity and yarn contact. Fabric density, particularly weft density, governs the structural alignment of yarns. A 35% increase in weft density (W19–W27) resulted in a 13–15% reduction in resistance, confirming that denser fabrics facilitate current flow. Higher weft density also increases directional resistance differences, enhancing anisotropic behavior. Angular distribution analysis showed lower resistance and greater anisotropy at perpendicular orientations (0° and 180°, the weft direction; 90° and 270°, the warp direction), while diagonal directions (45°, 135°, 225°, and 315°) exhibited higher resistance. Surface roughness further hindered current flow, whereas increased weft density and surface mass reduced resistance and improved the directional dependencies of the electrical resistances. This analysis was conducted based on research using woven fabrics produced from silver-plated polyamide yarns (Shieldex^®^ 117/17 HCB). These insights support the optimization of these conductive fabrics for smart textiles, wearable sensors, and e-textiles. Fabric variants W19 and W21, with lower resistance variability and better isotropic behavior under the *S* electrode arrangement, could be proposed as suitable materials for integration into compact sensing systems like heart rate or bio-signal monitors.

## 1. Introduction

The development of electronic textiles is rapidly increasing due to their ability to be integrated into clothing materials, offering a flexible and lightweight alternative to conventional electronic components [1,2]. The growing demand for conductive textiles is driven by the increasing adoption of smart textiles in various applications, such as wearable electronics, medical monitoring devices, and interactive clothing. The electrical performance of conductive woven fabrics is strongly influenced by surface characteristics such as topography, yarn distribution, and contact geometry [3,4]. These surface characteristics, alongside material composition, weave structure, and yarn alignment, all influence the electrical properties of woven fabrics. While surface characteristics may have a direct impact on conductivity, other factors, such as material composition and yarn alignment, determine the overall electrical performance and anisotropy of the fabric [5]. In addition, the surface properties of fabrics are critical for various functional applications, such as electromagnetic interference (EMI) shielding, thermal management, and personal protection. For instance, uniform electric current distribution on fabric surfaces can provide effective EMI shielding and thermal camouflage [6,7]. This interaction is also significant in applications such as touch-screen interfaces, medical sensors, and other electrode-based electronic devices for the efficiency of electrical signal transmissions [8,9].

Fabric surface measurement methods are vital in understanding and evaluating the texture and structural integrity of textile materials. These methods focus on quantifying the characteristics that contribute to the overall feel of a fabric, which is critical for both fabric designers and final users. In traditional textiles, “fabric handle” encompasses all the sensations experienced when touching a fabric. This handle is influenced by the fabric’s mechanical and surface properties and is crucial for consumers when assessing fabric quality and predicting the performance of a textile product. In the context of conventional textiles, precise measurement of surface properties enables researchers and fabric professionals to better understand the factors influencing fabric behavior. This knowledge enhances product functionality and improves the technical performance of textile materials, making them more suitable for applications such as wearable electronics [10,11,12,13]. Rougher surfaces, often resulting from higher fabric density or specific yarn structures, can reduce breathability by restricting airflow through the fabric. This is a critical consideration for textile materials, particularly when compared to traditional plastics and metallic components used in electronics. Textiles offer unique advantages, such as comfort and flexibility, making them highly suitable for wearable applications [14]. Another essential factor related to fabric surface profiles is the tactile feel, which is directly influenced by texture. Rougher fabrics can cause skin irritation upon contact, impacting user comfort [15,16]. Aesthetic appeal is also a significant factor, as the visual texture of roughness contributes to the fabric’s overall appearance and desirability [17]. Thermal insulation is another property significantly influenced by surface texture. The fabric’s ability to retain or dissipate thermal energy is directly affected by its micro- and macro-scale roughness, which in turn impacts thermal regulation properties such as heat retention or dissipation. These properties are critical for maintaining comfort in both conventional textiles and wearable electronics applications [15,18]. Furthermore, the surface roughness of conductive fabrics plays a pivotal role in their applicability to wearable electronics. Surface topography significantly affects surface resistance, interfacial adhesion, and yarn or fabric compactness—key factors influencing signal transmission efficiency. As a result, surface roughness emerges as a critical parameter for optimizing the performance of conductive textiles in smart wearable systems.

With recent advancements in wearable electronics and smart textiles, the surface electrical resistance of textile fabrics has become a critical property. Recent studies indicate that surface roughness significantly influences electrical properties, highlighting its crucial role in advanced textile applications [19,20]. One study investigated the influence of three surface modification techniques—flocking, layer-by-layer (LbL) deposition, and screen printing—on the electroconductive and biophysical properties of fabrics composed of cotton, polyester, and viscose [21]. While these modifications were found to enhance surface conductivity, the resulting changes in air permeability and thermal properties may influence suitability for prolonged skin-contact applications such as heart rate monitoring. These effects underscore the need for alternative approaches that balance conductivity with user comfort parameters. In this context, the present research explored non-coated, woven-integrated conductive fabrics as a potential route to achieving functional electrical performance while retaining fabric flexibility, breathability, and surface topographical characteristics.

The structural alignment and the roughness of the fabric’s surface directly affect the distribution and mobility of electrical charges, which is fundamental for the reliable transmission of data in smart textiles and wearable electronics. This property is particularly essential for emerging applications of screen-sensitive products, such as mobile technology, computing interfaces, and advanced display systems, where optimized conductivity and surface interactions are crucial for performance and functionality. As the demand for these innovative applications grows, understanding the relationship between surface roughness and electrical resistance becomes increasingly essential for optimizing fabric performance in various conductive-fabric-based wearable technology contexts. However, accurate measurement can be challenging because some current may penetrate from the surface into the material’s interior, affecting the readings. This highlights the need to investigate fabric surface resistance while considering the entire thickness of the material. In addition to the roughness of the surface, factors such as the compactness and distribution of the yarns over the surface of the fabrics can influence the surface values of electrical resistances [22,23]. The magnitude of electrical resistance and its directional dependencies in conductive fabrics present significant challenges in surface studies. These factors are critical to understanding and optimizing the performance of conductive textiles for advanced applications. Various methods and techniques have been developed to assess the roughness of textile materials [24,25]. The conventional method of evaluating fabric roughness depends on subjective techniques, including visual inspection and microscope analysis, which are both labor-intensive and time-consuming. The manual fabric surface inspection method usually results in significant variations in fabric property assessment, highlighting the need for more efficient and objective approaches. Various techniques have been developed to measure fabric surface properties. These methods provide insights into texture, smoothness, and other tactile features that define a fabric’s overall characteristics. Among these, surface roughness is one of the fundamental factors that can express the fabric property towards the product surface irregularity and the users’ feel [26,27].

A major step in fabric surface quality control came in the 1980s with the introduction of the Kawabata Evaluation Systems for Fabrics (KES-F), providing an objective measurement approach [28]. While the KES-F systems gained considerable attention, their high cost and complex procedures have prompted researchers to seek straightforward, faster, and affordable alternatives for fabric quality control [11,29,30]. The objective group also consists of contact and noncontact methods, and the contact methods of fabric surface measurement are exposed to fluctuation because of the friction between the measuring devices and the substrate surface. The noncontact technique, which is the measurement of roughness profiles taken from the light reflection [31]; modified optical multidirectional roughness meter (light reflections); and 3D surface scanning system with the image processing and fractal dimensions show more detailed information about the surface properties than the subjective one [32,33]. Even though noncontact surface roughness measurement methods and standardized procedures are not widely used in textile materials, various instruments are employed to study the geometric structure of fabric surfaces and demonstrate the influence of fabric parameters on this structure [34].

The roughness is the physical boundary distinguishing the entire workpiece from its surrounding environment. Roughness measures the texture of a fabric surface and is quantified by the vertical deviations of a surface from its ideal form. If these deviations are large, the surface is rough; if they are small, the surface is smooth. The surface profile can be categorized into roughness, waviness, and form [35]. A profilometer is one of the leading surface roughness measurement methods. The surface profilometer is a metrology instrument for topically characterizing a product’s uppermost layers. It is a multipoint form of measurement that considers a material’s primary form and the textural variations of its surface, typically characterized as dimensional factuality and the deviation of fibers on the fabric’s surface. These methods usually scan a surface with an incident light source and measure the reflective light to acquire information about the product’s surface topography. This metrological approach utilizes an optical technique grounded in the principle of noncontact chromatic measurement to assess surface characteristics accurately. It is often favored over contact-based surface profiling methods due to its minimal invasiveness and enhanced measurement speed. The rough surface can be measured using profile and areal methods. The profile method provides limited information, as it only captures a single line, which may not represent the entire surface. It measures surface roughness over a whole area, providing a 3D representation of the surface. The standard parameters, such as *sRa* (average roughness over an area), *sRz* (maximum height of the surface), and *sRq* (root mean square roughness), could represent the surface of the materials for an overall view of the roughness profiles [36].

In addition to the physical properties, the performance of smart textiles is influenced by their electrical properties, which affect the speed, capacity, and range of electronic data transmission in wearable electronics applications [37,38]. The fundamental goal in designing high-performance conductive textile fabrics is to minimize signal loss during the reception and transmission of electric current. A fast response time is crucial for the effective execution of targeted applications, such as in sensors and smart materials, in actual conditions. The response of the materials to electrical signals is directly related to the electrical resistance properties of the materials (fabrics), and the sensitivity can be determined from the measured values of resistance. Methods for measuring electrical resistance include (1) the two-electrode method, suitable for samples with large thickness; (2) the three-electrode method, which requires relatively large flat samples; (3) the four-electrode method, including the Van der Pauw technique, designed for measuring the electrical resistance of conductive materials with minimal thickness while minimizing the influence of contact resistance through a specific electrode arrangement; and (4) methods utilizing more than four electrodes, such as the Montgomery method, which involves six to eight contacts on the upper and lower parts of the sample [39,40].

The Van der Pauw (VdP) method was chosen for this research because it is suitable for measuring very thin or sheet-like conductive textile materials. This technique was initially designed to determine the electrical resistivity and Hall coefficient of flat, thin samples by placing four electrodes around the sample’s perimeter and conducting a series of current and voltage measurements. Current electrodes (two) inject a known current into the sample, while voltage electrodes (two) measure the voltage drop across them, and this separation eliminates the influence of contact resistance, ensuring accurate resistance measurements. The Van der Pauw method assumes material isotropy (uniformity in all directions). While the Van der Pauw (VdP) method is widely used for thin, isotropic films, its application to textiles and anisotropic materials needs implementation of combined approaches such as the four-point probe measurements and geometric averaging, and there are applicable initiatives to incorporate 3D finite-element modeling [39,41,42,43]. In textiles, fabrics often exhibit anisotropic behavior due to structural variations in their woven patterns, resulting in differences in electrical resistance along the warp, weft, and diagonal directions. This method enables the assessment of directional dependencies in electrical resistance under compression, which is not expected to follow isotropic behavior. Unlike the Montgomery method, VdP assumes isotropy and does not account for directional variation. This approach reveals resistance variability and highlights the combined effects of anisotropy. To fully characterize the fabric’s electrical properties, measurements in multiple directions are essential. Unlike traditional methods that require specific sample shapes, it can measure samples of arbitrary shapes, such as circular and rectangular shapes of materials [41,42,44]. Factors such as the raw material composition, the geometric properties, the alignment of the yarns, and the surface area between the electrodes determine the electrical resistance of textile materials.

The growing demand for electrically conductive fabrics in wearable applications faces significant challenges in achieving uniform current distribution, which is influenced by surface waviness caused by fiber deviation and the inherent directional dependencies of textile structures. Understanding how fabric density variations affect electrical resistance requires consideration of critical factors such as yarn orientation, angular distribution, fabric thickness, and surface mass. Establishing quantitative relationships between structural and surface roughness parameters and electrical conductivity is essential to ensure data reliability, skin-friendliness, and functional performance in wearable electronics. While prior studies have primarily focused on the mechanical and physical behavior of textile materials, the systematic analysis of surface irregularities, warp–weft density variability, and their combined effect on conductive behavior remains limited.

The impact of woven fabric structures on their electro-conductive behavior was the subject of research, and a novel anisotropy coefficient (*D%*) to evaluate directional resistance in woven fabrics was proposed [45]. Although the fabrics exhibited variable conductivity, uncontrolled variations in weave structure and fabric density led to anisotropic properties, highlighting undiscovered areas concerning the reproducibility and structural consistency of conductive woven fabrics. In particular, the quantification of surface irregularity (roughness profiles) and the distribution of these irregularities across the fabric surface (angular distribution) remains inadequately characterized. These factors are critical for sensitive applications, such as heart rate, respiratory, and pulse monitoring, in biomedical applications, where stable and isotropic signal transmission is essential. The main contribution of this paper is the systematic investigation of how structural parameters—specifically, surface profiles, weft density, surface mass, and fabric thickness—influence the anisotropic electro-conductive behavior based on woven fabrics constructed from silver-plated polyamide yarns. By refining the anisotropy coefficient and targeting balanced fabric structures with minimal directional variability (*D%* approaching zero), this study aims to advance the development of isotropic conductive fabrics optimized for reliable electrical properties, particularly for data monitoring in wearable electronics applications. This study employs polar geometric averaging and the Van der Pauw method, with a four-point probe electrode arrangement, to analyze electrical resistance in relation to directional conductivity, surface profiles, and geometrical parameters. These methodologies facilitate a comprehensive understanding of how structural and surface characteristics influence the electro-conductive performance of woven textiles produced for this research purpose. Previous studies have identified structural factors—such as fabric density, weave structure, and thread configurations—as key contributors to anisotropic electrical resistance within and between conductive fabrics [45]. This present study introduces a novel approach by integrating surface roughness profiling and angular yarn distribution analysis to assess their combined impact on the electrical anisotropy of plain-weave conductive fabrics. While previous research has acknowledged the role of structural factors such as fabric density and weave structure in influencing electrical properties, the specific effects of surface topography and yarn orientation on directional conductivity remain underexplored. By employing the Van der Pauw method for multidirectional resistance measurements and quantifying surface irregularities, we establish a direct correlation between surface profile variations and anisotropy coefficients (*D%*). This comprehensive analysis enables a deeper understanding of how structural and surface parameters affect the electro-conductive performance of textiles, providing valuable insights for the optimization of woven conductive fabrics in applications like wearable electronics, healthcare monitoring, and flexible technological devices.

## 2. Materials

The study investigated five types of electroconductive woven fabrics to evaluate the influence of fabric surface roughness and structural characteristics on their electrical behavior. Custom-designed fabrics were explicitly woven for this research, with controlled variations in the weft density of electroconductive yarns. Mechanical control of the take-up system was employed on Dornier m.b. to vary the weft density of the electroconductive yarns systematically. An H, GTN6/SD (Dornier Gesellschaft mit beschränkter Haftung) rapier weaving loom (Anl. no. 533684), equipped with a gear-based take-up control system to achieve variable weft densities, was utilized. The take-off motion of the loom was modified using six distinct gear configurations (gear pairs: 39/38, 43, 47, 51, and 55), each corresponding to a unique take-up rate. This mechanical modulation directly influenced the insertion rate of the weft yarns, resulting in five discrete weft densities: W19 (20.5 threads/cm), W21 (21.3 threads/cm), W23 (23.3 threads/cm), W25 (25.2 threads/cm), and W27 (27.6 threads/cm). The relationship between gear ratio and resulting weft density was established empirically and validated through optical analysis. Specifically, the actual number of warp and weft yarns per unit length was quantified using an Olympus SZX10 stereo microscope (Olympus Corporation, Tokyo, Japan) in combination with Stream Motion image analysis software version 1.5.1 (Olympus, Münster, Germany). Optical micrographs were captured for each sample, and the pick density was determined by averaging measurements from a minimum of five randomly selected microscopic fields on the samples. All other weaving parameters, such as warp tension, shedding sequence, and beat-up force, were held constant throughout the experiments to ensure that variations in structural properties could be attributed solely to changes in weft density. This methodology provided fine-tuned and reliable control over fabric architecture, enabling systematic investigation of its influence on the performance metrics of woven electroconductive textiles. To validate the achieved weft densities, microscopic inspection and manual pick counting were performed. This controlled fabrication process provided a reliable basis for analyzing how variations in electroconductive yarn concentration impact electrical resistance, surface topology, and anisotropic behavior of the woven structures.

**Figure 1:**
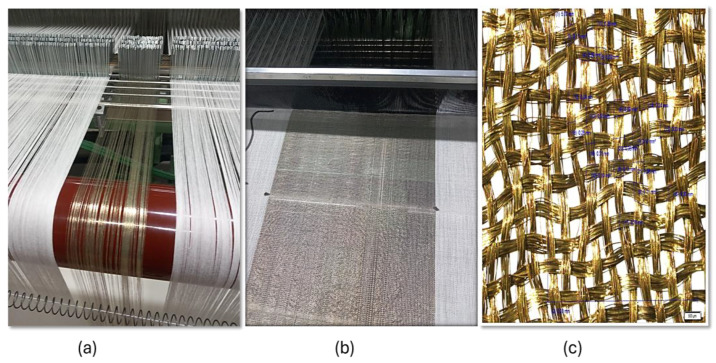
(**a**) The drawing-in warp thread under the loom machine; (**b**) the woven structure under the loom machine; (**c**) the developed plain-weave structure under the optical microscope.

Electrically conductive twisted silver-plated yarn, Shieldex^®^ 117/17 HCB, was used to produce plain-weave fabrics. According to the manufacturer’s technical information, the yarn was made from Polyamide (Nylon 6.6) as the base fiber, plated with 99.9% pure silver to enhance conductivity and provide antimicrobial properties. The yarn also has a nitrile rubber coating that is believed to provide additional protection against wear and environmental factors. After silverization, the raw yarn count is 117/17 dtex (+6/−3 dtex), increasing to 146 dtex (±6 dtex). It has an electrical resistivity of less than 500 Ω/m, ensuring conductivity, with a yield of approximately 68,000 g/m^2^ (±3%). The yarn strength is about tenacity exceeding 44 cN/tex and elongation at break of 26% (±6%). The samples were produced in the Weaving Technology Lab of the University of Borås in Sweden. In the fabric production process, the conductive warp yarns were drawn manually and introduced to the conventional loom machine, as shown in Figure 1.

Conductive yarns were manually integrated into the warp by replacing pre-drawn non-conductive warp yarns with conductive ones, as illustrated in Figure 1a. The same conductive thread was used for both warp and weft, with weft yarns inserted using conventional weaving techniques. Using identical conductive yarns in both directions ensured uniform structural and electrical properties across the fabric, thereby enhancing consistency in current distribution. This approach allowed for the seamless incorporation of conductive elements into the fabric while adhering to standard weaving practices. The woven structure under the loom machine is shown in Figure 1b. The actual number of warp and weft yarns was determined using an Olympus SZX10 Stereo Microscope (Olympus Corporation, Tokyo, Japan) and Stream Motion software version 1.5.1 (Olympus Corporation, Münster, Germany). The optical images of the structure are presented in Figure 1c. The structural parameters of the materials and the designations of samples are presented in Table 1.

The fabric parameters listed in Table 1 are as follows: *WaD*, *WeD*, *SM*, and *Th*, representing warp density, weft density, surface mass, and fabric thickness, respectively. *WaD* (warp density, expressed in threads/cm) refers to the number of warp yarns per centimeter, influencing the longitudinal stability and mechanical properties of the fabric. *WeD* (weft density, in threads/cm) denotes the number of weft threads per centimeter, which directly affects the concentration and distribution of electroconductive yarns in the fabric. *SM* (surface mass, in g·m^−2^) is the weight of the fabric per unit area, serving as an indicator of the fabric’s overall material density and structural compactness. *Th* (thickness, in mm) represents the fabric’s vertical dimension, which affects the depth of current penetration and the overall resistive behavior of the material. Table 1 presents the key structural parameters of plain-weave (1/1) fabric samples (W19–W27). As weft density increases from 20.5 to 27.6 threads/cm, surface mass and thickness also rise, indicating a trend toward a wider range of fabric density, thickness, and surface mass of fabrics used for this research.

## 3. Methods

### 3.1. The Fabric Surface Topography Determinations

The surface topography of woven fabrics was performed by means of the contactless method using MicroSpy^®^ Profile profilometer by FRT (Fries Research & Technology) Metrology™. The instrument uses an optical method based on chromatic distance measurement to analyze surface characteristics precisely. Its noncontact optical scanning technique captures high-resolution 3D images of surface topography with submicron accuracy. A built-in confocal microscope directs a beam of white light onto the sample surface and analyzes the reflected light to measure the height and shape of surface features [26,42]. For each fabric variant, scanning was conducted at the center of circular fabric samples over an area of 40 mm × 40 mm. The acquired surface scans were processed using Mark III software, version 3.11.4 (FRT Metrology™, Bergisch Gladbach, Germany), and the geometric structure of the fabric surface was assessed [32,33,46,47] following the ISO 4287 standard [48]. The key parameters analyzed in the surface roughness profile include *sRa* (arithmetic mean deviation, mm), *sRz* (maximum height, mm), *sRv* (valley depth, mm), *sRk* (core roughness depth), *sRsk* (skewness of the surface profile), and *sRku* (kurtosis). These parameters were investigated as the primary indicators for assessing the surface roughness characteristics in this study. The contact-free, non-destructive measurement system provided by FRT Metrology™ enabled high-precision profiling of surface features such as topography, step height, roughness, and surface thickness of the woven fabrics. The results were analyzed using the arithmetic mean height (*sRa*) in accordance with area-dependent roughness standards, with the following calculation:

(1) $$sRa=\frac{1}{A}\int \int_{A}|Z(x,y)|\;dx\;dy$$

where the parameter *sRa* is an arithmetic mean of the absolute of the ordinate values within a defined area of the fabric surface (*A*), which is the surface area being measured *Z*(*x*,*y*) and the height of the surface at the point (*x*,*y*) relative to the mean plane. This parameter helps to evaluate a surface’s roughness and extends the line-based *sRa* (arithmetically mean height) to a surface area of the woven structures. The parameter *sRz* in surface roughness testing represents the maximum height of the surface profile. In contrast, its transformed form, *sRz*25, represents the average of the maximum peak heights and valley depths over 25 evaluation lengths of the fabric. Unlike other measurements, *sRz*25 provides a more comprehensive understanding of surface texture, making it particularly valuable for applications that demand control of surface properties. The value of *sRz*25 is calculated using the following equation:

(2) $$sRz25\;=\;\frac{1}{25}\;\sum_{i=1}^{25}\left(Z_{p,i}\;+\;Z_{v,i}\right)$$

where *Z_p,i_* is the height of the twenty-five highest peaks, and *Z_v,i_* is the depth of the twenty-five deepest valleys, measuring the distance between the highest peak and the lowest valley in the specified section.

Parameter *sRsk* represents the skewness of the surface roughness and measures the asymmetry of the surface profile, indicating whether the surface contains more peaks or valleys, and it is given by the following formula:

(3) $${\;sR}_{sk}\;=\;\frac{\frac{1}{N}\;\sum_{i=1}^{N}{\left(Z_{i}\;-\;\bar{Z}\right)}^{3}\;}{{\left(\frac{1}{N}\;\sum_{i=1}^{N}{\left(Z_{i}\;-\;\;\bar{Z}\;\right)}^{2}\;\;\right)}^{3/2}}$$

where *Z_i_* represents the height value at the *i*-th point in the surface profile, $\bar{Z}$ is the mean height of the fabric surface, and *N* denotes the total number of height measurements in the woven fabric surface profile.

A positive skewness value suggests a surface dominated by peaks. In contrast, a negative value indicates a surface with more valleys [32,33,49]. These parameters collectively offer a comprehensive understanding of the fabric’s surface texture and roughness characteristics. The skewness parameter (*sRsk*) describes the asymmetry of the height distribution; when the distribution is skewed towards peaks, it indicates more upward features, whereas it signifies skewness towards valleys, reflecting more downward features of the materials surface and represent the symmetric height distribution. The value of zero represents a symmetric height distribution. The root means square roughness (*sRq*) provides a statistically weighted measure of surface height variations on textile fabrics, capturing both minor undulations and pronounced irregularities that influence yarn contact behavior and interfacial electrical performance, and it is given by the following equation:

(4) $$sRq\;=\;\sqrt{\frac{1}{N}\;\sum_{i=1}^{N}{\left(Z_{i}\;-\;\bar{Z}\right)}^{2}}$$

where *sRq* (root mean square roughness) represents the standard deviation of surface height variations from the mean. In textiles, it quantifies overall surface texture, where higher values indicate rougher fabrics, affecting comfort, friction, and sensor contact quality. Parameter *Z_i_* is the surface height at point, *Z_i_* is the mean surface height, and *N* is the number of data points.

Kurtosis (*sRku*) measures the sharpness of surface peaks and valleys, providing insight into the distribution of extreme values within the surface texture, and it is given by the following formula:

(5) $$sRku=\frac{1}{N\cdot s_{q}^{4}}\sum_{i=1}^{N}{\left(Z_{i}-\bar{Z}\;\right)}^{4}$$

where *sRku* is the surface kurtosis, *N* is the number of data points, *Z_i_* is the surface height at point *i*, $\bar{Z}$ is the mean surface height, and *s_q_*^4^ is the root mean square roughness (*sRq*), a statistical measure of surface height distribution sharpness. A platykurtic distribution indicates a flatter surface with fewer extreme values, while higher kurtosis signifies sharper peaks and more profound valleys.

The *Rmax*25 parameter represents the maximum peak-to-valley height within a defined sampling length of 25 points on the fabric surface, mathematically expressed as follows:

*Rmax*25 = max (Z_*peak*_ − Z_*valley*_) (6)

where *Z_peak_* is the height of the highest peak within the *Z_peak_* and the height of the highest peak within the *25*-point length*; Z_valley_* is the depth of the deepest valley within the 25-point length. This metric captures the most extreme topographical variation across the sampled region, making it particularly relevant for evaluating potential disruptions in contact continuity and localized increases in electrical resistance in electroconductive textile applications.

The angle distribution determination was conducted using profilometry, measuring surface profile angle (*θ*) and prominence (*p’*) across four quadrants (0–90°, 90–180°, 180–270°, and 270–360°). The angles represent the dominant orientation of surface features, while prominence quantifies their relative intensity as a percentage, reflecting the magnitude of surface texture variations. This method provides a comprehensive characterization of the fabric’s surface structural anisotropy and texture uniformity. The prominence (*p’*) values are typically expressed as percentages (%). These values represent the relative intensity or magnitude of the surface features (e.g., yarns, fibers, or texture) at specific angles (*θ*) across the four quadrants.

### 3.2. Electrical Resistance Measurement

The electrical resistance of the fabric samples was measured using the Van der Pauw method with a four-point probe electrode arrangement. Four cylindrical brass electrodes were positioned on the surface of each sample in a square pattern, with two electrodes injecting current and two measuring the voltage drop. While the Van der Pauw method assumes material isotropy, the anisotropic behavior of the fabrics, resulting from variations in resistance along the warp, weft, and diagonal directions, was accounted for by conducting measurements in multiple directions. This approach enabled accurate characterization of the fabric’s electrical anisotropy, addressing its inherent structural variations.

**Figure 2:**
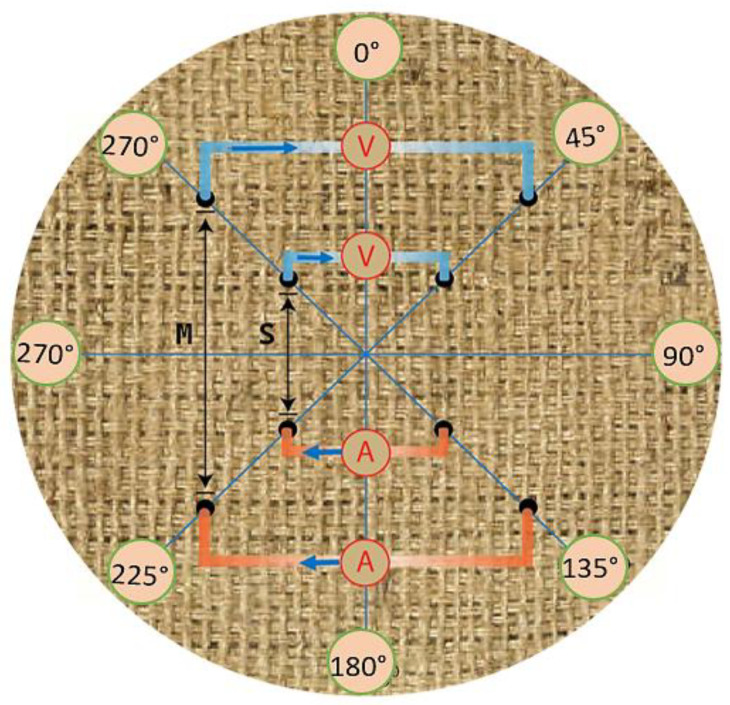
Electrode arrangement for *M* and *S* (medium and small electrode arrangement) on the sample plane.

Five electro-conductive plain-weave fabrics were prepared as circular samples, each with a diameter of 7 cm. Two electrode arrangements, *M* (medium) and *S* (small), were used, with spacings of 4 cm and 2 cm, respectively, between the electrodes on the textile substrate. The upper electrodes were connected to a voltage source (Agilent 34410A, Agilent Technologies, Santa Clara, CA, USA), while the lower electrodes were attached to a direct current (DC) source [41,50]. Measurements were conducted under controlled environmental conditions (41% relative humidity and 21.5 °C) to minimize the influence of external factors. Electrical resistance was calculated using Ohm’s law, and directional dependencies were evaluated based on the two electrode arrangements (*M* and *S*). Cylindrical brass electrodes with contact areas of 2 mm in diameter were arranged on the textile substrate in configurations spanning from 0° to 315°, as illustrated in Figure 2.

In the Van der Pauw method, a constant current of 0.04 A was applied to the two current electrodes, and voltage drop measurements were taken for eight different electrode configurations. These configurations were positioned at 45° intervals in a clockwise direction, starting at 0° and ending at 315°, completing a full circular rotation. Measurements were taken along the weft, warp, diagonal, and opposite directions by rotating the grouped electrodes over the pre-positioned circular fabric sample holder, as shown in Figure 2. The starting point, corresponding to the weft direction, was identified based on the orientation with the lowest observed voltage drops. To maintain consistent electrode contact, the fabric was rotated in 45° increments using a custom-designed holding mechanism while the electrodes remained stationary. This ensured accurate resistance measurements at each specified angle. The fabric remained stationary on a custom-designed, paper-based substrate throughout the process, minimizing potential sources of variability. The four electrodes (two uppers and two bottoms) were only moved up and down for fabric insertions.

**Figure 3:**
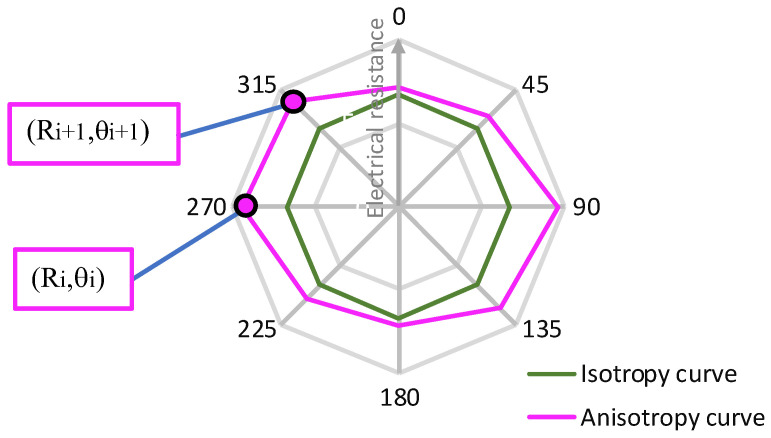
Curves represent the electrical resistance isotropy and anisotropy of fabric in polar coordinates.

Electrical measurements were performed using the Van der Pauw method in combination with a four-point probe electrode configuration. Each measurement was repeated five times to ensure accuracy, and the mean value was computed. The analysis of surface resistance was based on the geometric mean of measurements taken in polar coordinates, treating the circular textile samples as planar conductive surfaces [51,52], as illustrated in Figure 3.

The anisotropy curve is described using neighboring two-dimensional polar coordinates, denoted as (*R_i_*,*θ_i_*) and (*R_i_*_+1_*,θ_i_*_+1_), where *θ_i_*_+1_ − *θ_i_ >* 0 for *i =* 1,2, …, *n*, and *n* represents the total number of segments forming the curve (Figure 3). The length of the anisotropy curve was determined using the following formula:

(7) $$Daniso=\sum_{i=1}^{n}\sqrt{R_{i}^{2}+R_{i+1}^{2}-2R_{i}R_{i+1}\cos\left(\theta_{i+1}-\theta_{i}\right)}$$

The angle *θ_i_* is to be changed from *0°* to 315°. In the case of the isotropic sample, *R_i_*_+1_ *= R_i_* = *Rm* is obtained for *i* = 1,2, …, *n.* Based on Figure 3, the length of the polar isotropy curve becomes as follows:

(8) $$Diso=Rm\sum_{i=1}^{n}2\sin\left(\frac{\theta_{i+1}-\theta_{i}}{2}\right)$$

where *Diso* is the curve length of resistance for isotropic samples (*Rm*), and the mean resistance value can be expressed by the following formula [45]:

(9) $$\;Rm=\sqrt[{n}]{\prod_{i=1}^{n}R_{i}}$$

Assuming the constant angle increment *θ_i_*_+1_ − *θ_i_ =* 45° ∀ *i* = 1,2,…,8, the length of the isotropy curve can be expressed as given:

(10) $$Diso=16Rm\;\cdot \;\sin\left(\frac{\theta_{i+1}-\theta_{i}}{8}\right)\;$$

(11) Diso=6.12Rm

where *Rm* is the isotropic resistance from the experimental result according to the electrode configuration arrangement illustrated in Figure 2 and calculated according to Equation (9). The parameter *D%* was used to measure the electrical in-plane anisotropy of conductive woven fabrics, which determines the relative difference in the length of the anisotropy and isotropy curves. The anisotropy coefficient *D%* is given as follows [45]:

(12) $$\;D\%=\frac{Daniso-Diso}{Diso}100\%$$

A value of *D%* equal to zero indicates that the fabric exhibits isotropic electrical properties. The higher the value of the anisotropy coefficient, the higher the electrical resistance directional dependency of the fabrics.

In the characterization of anisotropic properties (*D%*) and electrical resistance (*Rm*), the electrodes configured at angles ranging from 0° to 315° play a crucial role in analyzing the electrical properties of the fabric surface, as they make direct contact with the material to collect electrical conductivity data. The flow of electric current is closely linked to the surface’s irregularity and compactness, which provides insights into the ease of current passage. The results were analyzed based on two key parameter categories: the fabric surface roughness profile and the angular distribution of the yarns. These analyses offer essential insights into how surface characteristics such as roughness and yarn orientation influence the electrical behavior of the fabric, establishing a clear connection between structural properties and performance.

## 4. Results and Discussion

### 4.1. Surface Roughness Profile Analysis

**Figure 4:**
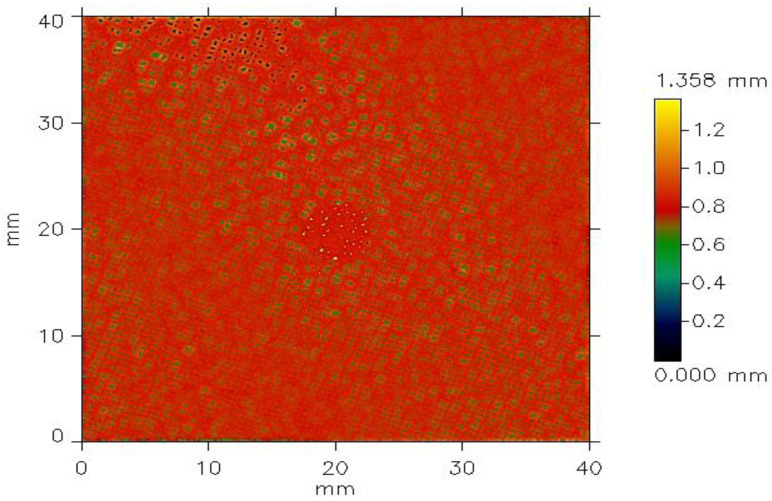
Surface profile plot for sample W19.

**Figure 5:**
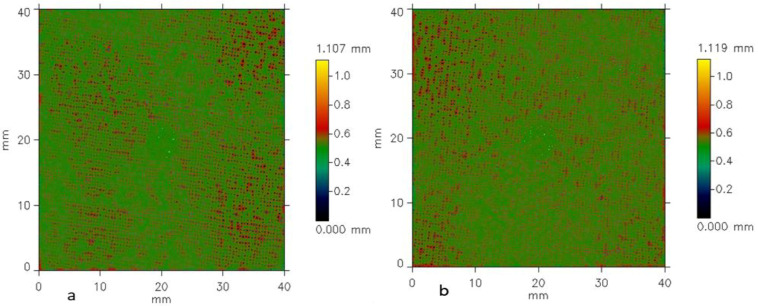
Surface profile plot for samples: (**a**) W21*;* (**b**) W23.

**Figure 6:**
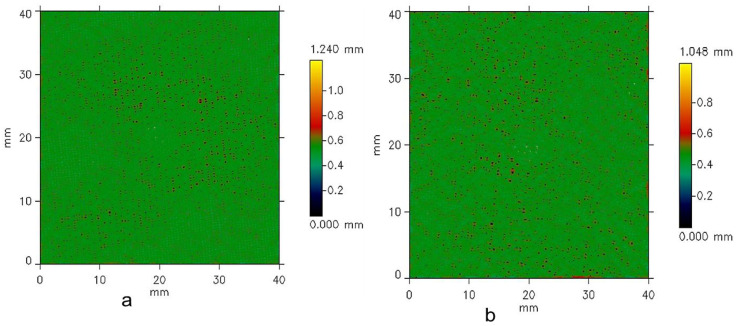
Surface profile plot for samples: (**a**) W25; (**b**) W27.

A surface roughness analysis was performed on plain-weave fabric samples using a non-contact optical profilometer. Surface scans were captured and analyzed with dedicated software in accordance with the ISO 4287 standard [48]. Figure 4, Figure 5 and Figure 6 display the results of the surface roughness analysis, while Table 2 summarizes the corresponding roughness profile values. In the context of surface roughness and profilometry measurements, *Ls* (sampling length) and *Lc* (cut-off length or filter length) are parameters related to the analysis and filtering of the surface profile data. *Ls* refers to the surface length over which the measurement is taken, representing the distance along the surface over which data points are collected to analyze the roughness profile. In this study, *Ls* indicates that the measurement was taken over a *Ls* = 245.0 µm length of the fabric surface. *Lc*, on the other hand, is a filtering parameter that separates the roughness profile from the waviness profile. The cut-off filter (*Lc*) was applied to remove the waviness component from the primary profile. By filtering out the long-wavelength elements associated with waviness, a roughness profile was generated, which isolates the finer surface texture details. It determines the threshold between roughness (shorter wavelengths) and waviness (longer wavelengths) on the surface of the fabrics. With *Lc* = 0.800 mm, features smaller than *Lc* are considered part of the roughness, while more prominent features are classified as waviness. These parameters are essential for accurately characterizing the surface texture and understanding the distinction between fine roughness and broader waviness in the fabric samples. The results of the structural properties and surface roughness analysis were performed according to the plain-weave fabric made from Shieldex^®^ 117/17 HCB samples using a non-contact optical profilometer. This effect is primarily due to the greater number of yarns per unit length, which enhances surface uniformity by minimizing air gaps and reducing the occurrence of loose or uneven threads. Moreover, higher yarn density typically induces greater yarn tension during the weaving process, promoting more consistent alignment and tighter integration of the yarns, which further contributes to a smoother fabric surface. This denser arrangement minimizes gaps between the yarns, leading to a smoother surface, as the yarns are less likely to protrude. Additionally, the consistent interlacing pattern of the weave creates a uniform texture, further contributing to a reduction in irregularities. The overall effect is a fabric that feels smoother and more refined with higher weft density. The relationship between weft density and the roughness of fabrics differs due to the weave’s unique structure. The divergence in weft and warp density is a more complex interlacing pattern that involves warp and weft yarns with varying thicknesses and spacing. As weft density increases, the weave’s distinctive texture and visual characteristics can become more pronounced, leading to a potential exhibit of unidirectional property. The interplay between thicker and thinner yarns can create irregularities that contribute to surface texture, and higher density does not necessarily result in fabrics with uniform geometrical and electrical properties. The profilometry method is a technique used to measure and analyze the surface profile of fabrics by scanning the surface using a non-contact chromatic white light sensor. This method records height variations (*Y*) as a function of distance (*X*), generating a detailed profile of the surface’s peaks and valleys. The collected data are then plotted as a 2D graph, with the *X*-axis representing the distance along the surface and the *Y-*axis representing the height or depth of the surface features. However, the structural complexity of the woven fabrics introduces variations in surface electrical resistance, which are not solely dependent on changes in weft density, as detailed in Table 1. The surface roughness profiles of the woven structures, measured with a sampling length (*Ls*) of 245.00 µm and a cut-off length (*Lc*) of 0.80 mm, are shown in Figure 4, Figure 5 and Figure 6. The *X* and *Y* axes indicate surface dimensions in mm, while the color scale represents the surface height in mm.

In the surface maps (Figure 4, Figure 5 and Figure 6), the color bar shows surface height, ranging from 0.00 mm (blue/green) to 1.358 mm (yellow/red). Sample W19 (Figure 4) had the highest surface roughness, with red areas and scattered spots indicating peaks or raised regions. Yellow areas correspond to the highest points, approaching the maximum height range. The sample surface in Figure 5a,b appears to show moderate uniformity, as less dominant red regions suggest the spread of roughness to different directions, with fewer smooth or flat areas. The yellow patches show the highest points distributed across the surface. These peaks indicate raised features such as fibers, yarn intersections, and general surface irregularities of the woven structures.

In the case of W25 and W27 in Figure 6, the green areas dominate the surface, suggesting the overall surface is relatively flat or uniform in height before reaching 0.6 mm. Blue or darker green areas were not observed in the case of these samples (in Figure 6a,b), denoting less availability of depressions or valleys on the surface of these samples. The green regions dominate the surface, indicating that most of the surface is relatively flat and uniform in height among the denser fabrics. Small black and blue spots scattered across the surface represent localized valleys. There are very low yellow regions that are minimal, suggesting very few areas approach the maximum height of 1.240 mm. Surface roughness parameters provide critical insights into the texture and functional properties of a surface, which are directly relevant to the electrical resistance of conductive fabrics. *sRa* and *sRq*, representing the average and root mean square roughness, respectively, indicate the overall smoothness or variability of the fabric surface. Smoother surfaces (lower *sRa* and *sRq*) typically result in better contact between conductive fibers, reducing electrical resistance. *sRz*25, which quantifies peak-to-valley heights, highlights the depth of surface irregularities that can disrupt electrical pathways, increasing resistance*. sRsk* (skewness) indicates whether the surface has more peaks or valleys, influencing contact points between fibers. At the same time, *sRku* (kurtosis) describes the sharpness of these features, which can impact the uniformity of the current distribution. These parameters are essential for optimizing the design and performance of conductive fabrics in applications like wearable electronics, sensors, and EMI shielding. A summary of typical values for these parameters is presented in Table 2.

Table 2 presents the surface roughness and electrical properties of conductive fabrics with varying fabric parameters, such as weft densities (from W19 to W27). Table 2 presents surface roughness parameters (*sRa, sRq, sRz25, sRsk*, and *sRku*) and electrical resistance measurements (*Rm M*, *Rm S*, *D%M*, and *D%S*) for five fabric samples (W19–W27). Regarding surface roughness, the *sRa* and *sRq* values decrease from W19 to W27, indicating a smoother surface in sample W27. Similarly, the reductions in *sRz*25 and *Rmax*25 suggest the presence of fewer and less pronounced surface peaks and valleys.

Parameter *sRmax*25 shows a notable decrease after the first sample, followed by relatively stable roughness values across the remaining fabrics. The *sRsk* values are highly damaging, indicating surfaces dominated by valleys rather than peaks. The *sRku* values are very high, especially for W25 and W27, suggesting sharp peaks and extreme surface variations. Regarding electrical resistance, *Rm* for both *M* and *S* electrode arrangements decreases from W19 to W27, correlating with reduced surface roughness. *D%M* and *D%S* increase, indicating greater structural changes in samples with smoother surfaces. Smoother surfaces (lower roughness values) generally exhibit lower electrical resistance, likely due to better fiber-to-fiber contact and reduced disruptions in conductive pathways. The high *sRku* and negative *sRsk* values suggest that surface irregularities, though reduced, remain sharp and valley-dominated, which may still impact conductivity. Comparing the surface profiles, the samples (W25 and W27) show lower roughness values and demonstrate improved conductivity and greater electrical anisotropy, as shown in the values provided in Table 2. These findings are critical for optimizing conductive fabrics for applications in wearable electronics and smart textiles.

**Figure 7:**
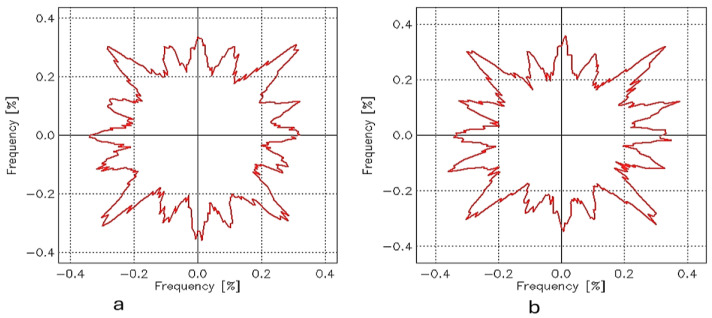
Angular distribution of the surface roughness for sample: (**a**) W19; (**b**) W21.

**Figure 8:**
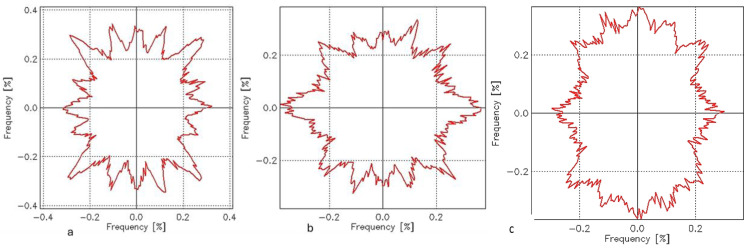
Angular distribution of the surface roughness for sample: (**a**) W23; (**b**) W25; (**c**) W27.

The surface roughness through the area of the samples is related to the orientation of the fibers over the surface of the fabrics. The angular distribution of surface roughness profiles was analyzed using a technique that measures the orientation of surface angles (*θ*) and their relative prominence (*ps*) across four quadrants. This method generates profiles of the dominant surface orientations, highlighting the direction and intensity of surface features. The results are represented in a quadrant-based plot, where angles indicate the direction of surface features and prominence values reflect their relative intensity. In surface roughness analysis, the angle (*θ)* represents the dominant orientation of surface features, measured in degrees (°), indicating the direction of peaks and valleys relative to a reference axis. The relative prominence (*ps*), expressed as a percentage (%), quantifies the intensity or significance of these features at specific angles, reflecting their contribution to the overall surface texture. These parameters provide valuable insights into the anisotropic behavior of the fabric surface, helping to assess texture uniformity and directional surface properties; the angular distribution of the surface roughness for the samples from W19 to W27 is presented in Figure 7 and Figure 8.

In the case of samples W19 (Figure 7a), W21 (Figure 7b), and W23 (Figure 8a), the angles show diagonal orientations close to 45°, 135°, 225°, and 315°, which is typical of the structural consistency of plain-weave fabrics with balanced warp and weft yarns. In contrast, W25 (Figure 8b) exhibits orientations closer to 0° and 180°, suggesting alignment with the warp and weft directions. Meanwhile, W27 (Figure 8c) displays orientations closer to 90° and 270°, indicating alignment perpendicular to the reference direction. Regarding relative prominence, *ps* has the lowest values across all quadrants, suggesting that its surface features are less pronounced or less aligned than those of the other samples. On the other hand, W19, W21, and W23 show higher *ps* values, with W21 being the most prominent. Table 3 presents the measured values of (*θ*) and (*ps*), highlighting the predominant orientations and their corresponding prominence levels.

These findings from Table 3 show that the variations in surface texture and alignment across the samples reflect differences in their structural and surface roughness properties. The analysis of angle distributions and relative prominence (*ps*) reveals the variations in surface texture and alignment across the fabric samples (W19, W21, W23, W25, and W27). These variations reflect differences in their structural properties and surface roughness, which are closely connected to fabric thickness, surface mass, and the weft density of the fabrics. Samples with higher prominence values (e.g., W19 and W21) exhibit more pronounced surface features, indicating rougher textures. This roughness creates air gaps and discontinuities in the conductive pathways, leading to higher electrical resistance. Samples with lower prominence values (e.g., W25 and W27) have smoother surfaces, resulting in better fiber contact and lower electrical resistance.

### 4.2. Electrical Resistance Analysis

**Figure 9:**
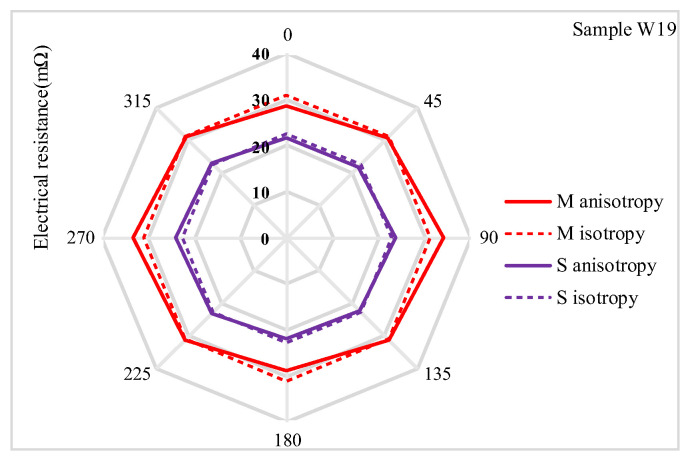
Isotropy and anisotropy curves for sample W19.

**Figure 10:**
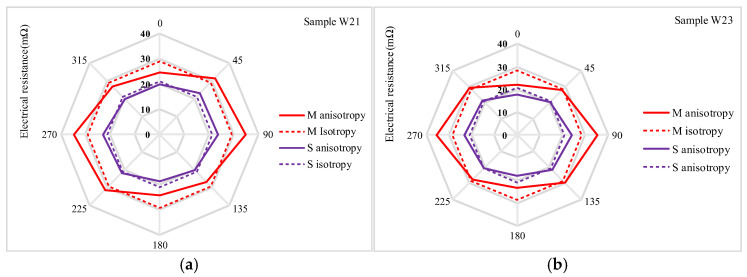
Isotropy and anisotropy curves for samples (**a**) W21; (**b**) W23.

**Figure 11:**
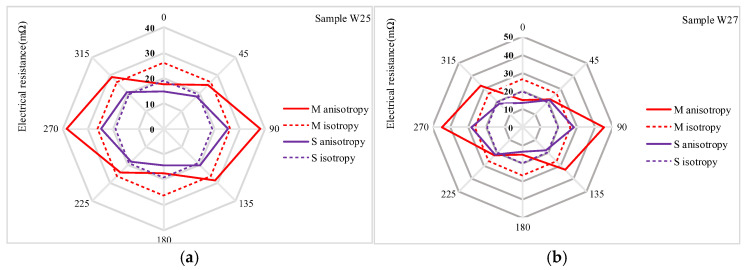
Isotropy and anisotropy curves for samples (**a**) W25; (**b**) W27.

The electrical resistance measurements obtained using the Van der Pauw method revealed electrical anisotropic properties in the two electrode arrangements, *M* and *S*. Rougher fabric surfaces, such as W19, W21, and W23, exhibited higher electrical resistance compared to denser fabrics like W25 and W27. This increase in resistance for the rougher fabrics is attributed to the creation of air gaps, which reduce the quality of contact between yarns and hinder the flow of electric current. The electrical anisotropy of woven fabrics is influenced by their structural orientations and the quality of yarn contacts within the direction of the fabric surface. Electrical conductivity varies depending on the direction of current flow relative to the weave patterns, resulting in different resistance values along the warp and weft directions. Additionally, the diagonal and other surface directions of the fabrics contribute to the variability in current flow. The warp and weft densities further explain these observations. The warp density ranges from 19.8 to 20.7 threads/cm, while the weft density varies from 20.5 to 27.6 threads/cm. The variation in weft density, while maintaining a relatively constant warp density (as shown in Table 1), underscores the influence of fabric structure on electrical properties. The anisotropic curve exhibits variability across the plane, whereas the isotropic curve remains constant from 0° to 315° in the electrode configuration represented in the radar charts (Figure 9, Figure 10 and Figure 11). The greater variability of the anisotropic curve compared to the isotropic curve in both *M* and *S* electrode arrangements indicates a higher anisotropic behavior of the fabrics in electrical resistance. The isotropic and anisotropic properties of the fabrics W19–W27 are presented in Figure 9, Figure 10 and Figure 11.

Figure 9 presents a comparison of electrical resistance (*Rm*) and anisotropy coefficients (*D%*) for fabric sample W19 in both (*M*) and (*S*) electrode arrangements. The isotropic and anisotropic measurements indicate that sample W19 behaves as an isotropic fabric, exhibiting electrical resistance values of 190.51 mΩ in the isotropic direction (*Diso*) and 191.67 mΩ in the anisotropic direction (*Daniso*). The minimal difference between these curves, encompassing total resistance values of isotropic and anisotropic resistances across the complete electrode configuration (0° to 315°), suggests that the fabric exhibits negligible anisotropic properties. The anisotropy coefficient (*D%*) is 0.6% for *M* and 0.2% for the *S* electrode arrangement, respectively, indicating that sample W19 has lower anisotropic properties. Additionally, the smaller fabric area within the four electrodes (*S*) shows fewer directional dependencies. Figure 10a compares the resistance of W21 for the *M* and *S* electrode arrangements. In this sample, the isotropy and anisotropy curves show greater variability than W19. The anisotropy coefficient (*D%*) increases to 3.6% for the *M* electrode arrangement and 1.2% for the *S* electrode arrangement. Additionally, the electrical resistance (*Rm*) decreases from 31.11 mΩ to 29.19 mΩ for the *M* electrode arrangement and from 22.68 mΩ to 21.19 mΩ for the *S* electrode arrangement. The anisotropy coefficient (*D%*) and electrical resistance Rm continued to decrease in sample W23 (as shown in Figure 10b for the transition from *M* to *S* electrode arrangement). This trend was continued across all samples, from W19 to W27. The highest anisotropy coefficient (*D%*) was observed in W27, with values of 29.9% for the *M* (medium) electrode arrangement and 13.7% for the *S* (small) electrode arrangement. Additionally, as shown in Figure 11a,b, the variability between the anisotropy and isotropy curves is also observed to increase, with the curves diverging further apart from each other (Table 4). Min–max normalization was applied to calculate the normalized electrical resistance (*Rm*) values for both *M* and *S* electrode arrangements. The values of the anisotropic and isotropic resistance are presented in Table 4. Min–max normalization was applied to calculate the normalized electrical resistance (*Rm*) values for both *M* and *S* electrode configurations.

Table 4 presents the electrical resistance (*Rm*), isotropic resistance over the total length of the curve (Figure 9, Figure 10 and Figure 11) (*Diso*), anisotropic resistance (*Daniso*), and anisotropy coefficient (*D%*) for five fabric variants (W19, W21, W23, W25, and W27) under two electrode arrangements (*M* and *S*). As shown in Table 4, sample W19 exhibits the highest normalized *Rm* under both configurations, while W25 has the lowest, reflecting relative resistance behavior independent of absolute values. These data highlight the influence of fabric parameters such as weft density on electrical properties and anisotropy. In all samples, the anisotropy coefficient (*D%*) increases as the fabric parameters, particularly weft density, change from W19 to W27. This trend indicates that structural modifications in the fabric, such as increased weft density, significantly impact electrical anisotropy. The observed electrical isotropy in W19 fabric samples can be attributed to the nearly identical warp and weft densities as well as the use of the same yarn type in both directions. This symmetric construction results in uniform electrical pathways, minimizing directional dependence on resistance. Notably, electrical resistance (*Rm*) and anisotropy (*D%*) show a decreasing trend when transitioning from the *M* to the *S* electrode arrangements. These values were measured along multiple directions, from 0° to 315°, following predetermined angular intervals. While the intrinsic electrical properties of the material remain unchanged, these measurements reflect how variations in fabric sizes and positioning relative to the predetermined configuration, such as weft, warp, or diagonal direction, can influence the observed resistance values. This is particularly relevant for anisotropic and inhomogeneous materials, where directional sensitivity plays a significant role. The behavior observed in sample W27, which exhibits a higher degree of anisotropy, further illustrates this effect. While an increase in weft yarn density generally leads to reduced electrical resistance and potentially higher anisotropy due to structural directionality, the observed variations suggest that other factors, such as yarn twist, evenness, and contact quality, also influence electrical behavior. Future studies will consider these aspects to clarify their role in anisotropic performance further.

A detailed analysis revealed that a 35% increase in weft density (from W19 to W27) resulted in a 13–15% reduction in electrical resistance, demonstrating that denser fabrics promote better current flow. This improvement in conductivity is likely due to the enhanced contact between fibers and the more uniform distribution of conductive pathways in denser fabrics. Furthermore, the anisotropic property (*D%*) for both *M* and *S* arrangements shows a significant surge from W19 to W27. Specifically, *D%* increases by +4900% for the *M* arrangement and +6750% for the *S* arrangement. This substantial amplification of the anisotropy ratio indicates that higher weft density not only improves conductivity but also intensifies the directional dependence of electrical properties. The S arrangement exhibits a more pronounced increase in anisotropy, suggesting that this configuration is more sensitive to changes in fabric structure. These findings underscore the intricate relationship between electrical properties and physical parameters such as weft density. Because of the weft density increments, the surface mass (*SM*, g·m^−2^) of the fabrics is pronounced, and it also affects the parameters *Rm* and *D%*. As *SM* increases, *Rm M* generally decreases, indicating better conductivity. For sample W19 with a surface mass of 60 g· m^2^, the electrical resistance (*Rm*) for the *M* electrode arrangement is 31.11 mΩ. In comparison, sample W27, with a surface mass of 75 g·m^−2^, shows an *Rm* value of 26.42 mΩ for the same electrode configuration. This suggests that higher surface mass improves conductivity, likely due to increased material availability for charge transport. *D%* increases significantly with *SM*, indicating greater directional dependence (anisotropy) in electrical properties. Sample W19, with a surface mass of 60 g· m^2^, exhibits a directional difference (*D%*) of 0.6%, indicating low anisotropy. In contrast, sample W27, with a surface mass of 75 g·m^2^, shows a *D%* of 29.9%, reflecting high anisotropy. This implies that higher *SM* leads to more pronounced differences in electrical resistance directional dependencies, which are due to an imbalance of fiber alignment or uneven material distribution on the fabric surface.

**Figure 12:**
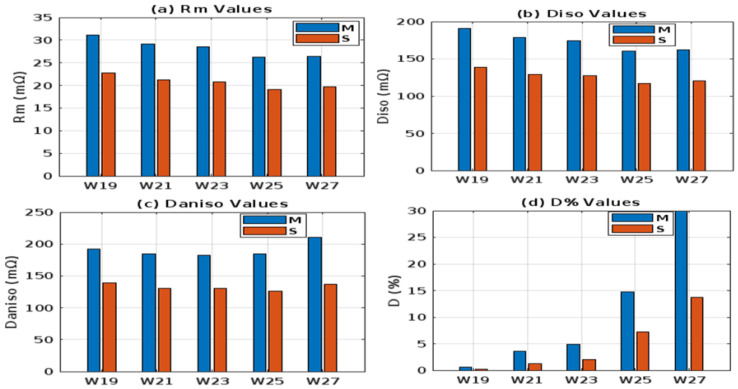
Change comparisons in electrical properties of the samples based on the magnitude of each values: (**a**) electrical resistance (*Rm*); (**b**) isotropic electrical resistance (*Diso*); (**c**) anisotropic electrical resistance (*Daniso*); (**d**) anisotropy coefficient (*D%*).

The overall trends are visually summarized in Figure 12, which illustrates how changes in fabric dimensions under the varying electrode configurations from *M* to *S* influence electrical resistance and anisotropy. This analysis provides valuable insights into the design and optimization of conductive fabrics for applications requiring specific electrical properties.

A consistent and gradual decrease in electrical resistance (*Rm*) was observed for both *M* and *S* configurations (Figure 12a). While a similar decreasing trend is evident in the degree of isotropy, i.e., *Diso* (Figure 12b), the variations are more pronounced when comparing *Diso* values across different fabric samples. Conversely, the extent of electrical anisotropy (*Daniso*) exhibits an increasing trend across all electrode arrangements, both within individual fabrics and between different groups of fabrics (Figure 12c). A significant disparity is observed in the degree of anisotropy (*D%*) (Figure 12d), indicating a substantial influence of anisotropic properties on the observed changes. Additionally, the fabric parameters and surface profile of the woven fabrics were analyzed as factors influencing their conductivity. A correlation analysis was conducted to determine if there is a direct relationship between the selected roughness profiles and electrical resistance (*Rm*) and *D%*. Pearson’s correlation coefficients, which were analyzed using PQStat software (version 1.8.6, 2023), are presented in Table 5. Coefficients with a significance level of the *p*-value < 0.05 are marked in bold.

Table 5 presents the Pearson correlation coefficients (*r*) between the surface roughness parameters (*sRa* (mm), *sRq* (mm), *sRz*25 (mm), and *sRmax*25 (mm), *sRsk, sRku*) and the electrical resistance measurements (*Rm* (mΩ) and *D%* (%), for both *M* and *S* electrode arrangements). Statistical analysis was performed using PQStat software (version 1.8.6, 2023). Correlations with statistical significance (*p*-value < 0.05) are highlighted in bold. Effect sizes were interpreted based on the criterion ∣*r*∣ ≥ 0.85, indicating a strong and statistically significant correlation. The arithmetic mean roughness (*sRa*) and root mean square roughness *(sRq*) show substantial positive correlations with electrical resistance (*Rm M* and *Rm S*). For instance, *sRa* and *Rm M* have a correlation coefficient of 0.9654, while s*Rq* and *Rm M* show an even stronger correlation of 0.9847. The observed trend indicating that increased surface roughness (*sRa* and *sRq*) corresponds to higher electrical resistance is based on the specific yarn and fabric configurations used in this study. This relationship is likely influenced by the amount and distribution of conductive yarn, which may vary with the different physical properties of the yarns and fabric parameters. As such, this analysis should not be generalized beyond the scope of the materials tested, which are plain-weave fabrics produced from Shieldex^®^ 117/17 HCB. Further research involving a broader range of conductive yarns and fabric structures is necessary to validate the general applicability of these findings.

The increased resistance is attributed to the loose structure, air gaps, and poor fiber-to-fiber contact caused by surface irregularities, which hinder the flow of electric current. Conversely, smoother surfaces (lower *sRa* and s*Rq*) exhibit lower resistance, as they provide better contact between conductive fibers and more uniform current flow. The anisotropy coefficient (*D%*), which measures the directional dependence of electrical properties, shows extensive negative correlations with surface roughness parameters. For example, *sRa* and *D*%*M* have a correlation coefficient of −0.9835, and s*Rq* and *D%M* show a correlation of −0.9670. This means that increasing roughness leads to decreasing anisotropy. Rougher surfaces introduce less variability in current flow, reducing the directional dependence of electrical properties. In contrast, smoother surfaces (lower *sRa* and *sRq*) exhibit higher anisotropy, as they allow for a more uniform and directional flow of electric current. Correlation analysis revealed that s*Rmax2*5 significantly affects electrical resistance only under the *S* electrode arrangement, while other surface parameters, such as *Rm M* and *D*%, show no notable impact in either configuration. These findings suggest that *sRmax*25, due to its shorter scan length and localized measurement scope, is less sensitive to surface anisotropy and more relevant for small-scale resistance measurements. The study underscores the importance of targeted surface analysis in predicting the electrical performance of textile-based conductors.

The electrode arrangement was changed from *M* to *S*, reducing the surface area of the fabrics. This adjustment resulted in less roughness due to smaller irregularities, while the number of warp and weft yarns, their interlacing, and the looseness of the threads were also reduced. This narrowing of the fabric’s structure impacted the electrical resistance (*Rm*). This was due to fewer threads and thread contacts, which maintained the fabric’s smoothness and allowed for a uniform flow of electric current across the surface of the fabric. The fabric’s surface area increased, allowing for the growth of rough surface deviations. This led to a high value of the electrical anisotropy coefficient *D%* of the fabrics. Plain-weave fabrics are constructed in a textile weave where warp and weft threads are interlaced in a crisscross pattern, resembling the deviation structure to create surface irregularity that can affect the current flow. The Mann–Whitney U (M-W.U) test in Table 6 was used to compare two groups of the electrode arrangement *M*-*S* to the electrical resistance *Rm* and anisotropy coefficient *D%*. To test the null hypothesis, a *p*-value < 0.05 was adopted for significant differences in the electrode arrangement and electrical properties.

**Figure 13:**
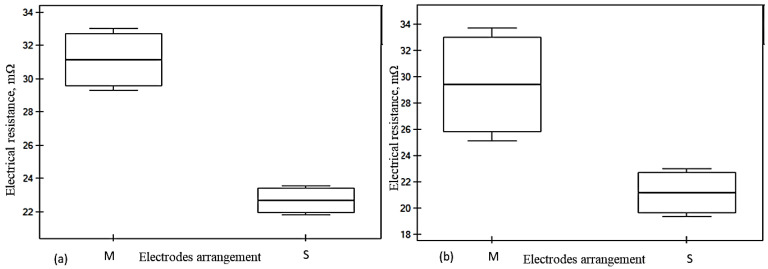
Comparison of the electrical resistance between the electrode arrangements (*M* and *S*) of the woven fabric samples: (**a**) W19; (**b**) W21.

**Figure 14:**
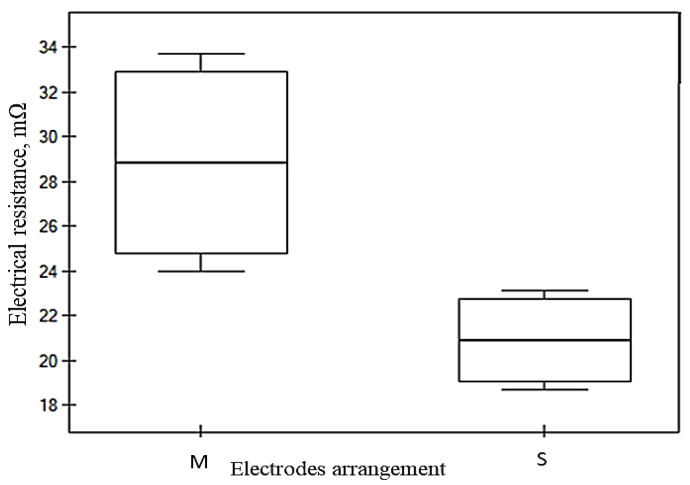
Comparison of the electrical resistance between the electrode arrangements (*M* and *S*) of the woven fabric sample W23.

The Mann–Whitney U test examines the statistical significance of electrode arrangement *M*-*S* to the electrical resistance (*Rm*) across the five conductive fabrics. In the case of W19 and W21, the highest *Z* statistics were demonstrated (3.310 and 3.308, respectively). This was also shown by highly significant results, with *p*-values far below the standard threshold of 0.05. In contrast, samples W25 and W27 exhibit weaker statistical significance, with effect sizes and *p*-values reflecting less pronounced differences. The key fabric parameters, which are presented in Table 6, namely weft density, mass per unit area, and fabric thickness, significantly influence electrical resistance (*Rm*) and anisotropic properties (*D%*). A significant difference was observed in the *Rm* values of W19, W21, and W23, indicating that the electrode arrangement (*M*-*S*) influenced the resistance values of the respective samples. Their relationship with the electrical resistance between samples was also examined in the *r*-Pearson correlation analysis. The significant correlation coefficients and their significance level are marked in bold and presented in Table 7. The two groups of electrode arrangements are represented in *M* and *S* in the box plot graph (Figure 13 and Figure 14). The greater spread in group *M* indicates that the electrical resistance within this group is more diverse than in group *S*.

The electrical resistance in the sample in Figure 13a, i.e., W19, with *M* to *S* arrangement, was characterized by higher variability (reduced by 27.12%) due to the availability of air and looseness of the yarns connected to the low weft density in the *M* electrode arrangement, as shown in Figure 13a. In the case of Figure 13b, i.e., W21, there is less variability in *Rm* for the *M*-*S* configuration, indicating more consistent and fewer paths for current flow. This trend continued to the denser fabrics, suggesting that the increase in the number of threads and their contact points significantly influences the rising trends of current flow over the fabric surface.

**Figure 15:**
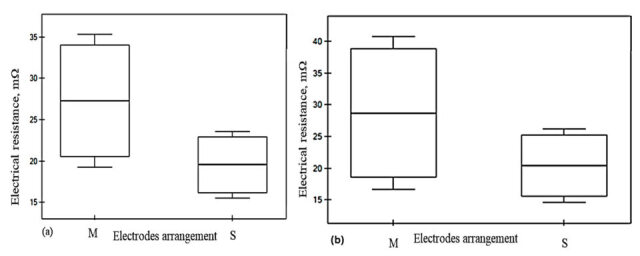
Comparison of the electrical resistance between the electrode arrangements (*M* and *S*) of the woven fabric samples: (**a**) W25; (**b**) W27.

In sample W23 (Figure 14), a noticeable trend is demonstrated with respect to changes in the fabrics under the four-electrode arrangement from *M* to *S*. This trend continues in W23, where Rm increases alongside fabric density; as illustrated in Figure 14, the resistance variability between the two electrodes is reduced by 26.89%. Additionally, fabrics with smaller widths within the four-electrode configuration exhibit reduced electrical resistance and anisotropy coefficient, as discussed previously in Figure 12a,d. The high weft density fabrics shown in Figure 15a,b (W25 and W27) exhibit significant electrical resistance; however, no significant variation is observed as a result of the electrode arrangement under the four-electrode (*M*-*S*) arrangements. This suggests that increasing fabric density may enhance the stability of electrical resistance due to dimensional consistency under defined electrode contact. Nevertheless, the imbalance between warp and weft densities, along with the electrical anisotropy, should be considered as influencing factors in further analysis. The influencing factors of the variability of electrical resistance and anisotropy are discussed in Table 7.

The variables (*WaD*), (*WeD*), (*SM*), and (*Th*) represent the warp (*WaD*) and weft (*WeD*) densities in threads/cm, surface mass (*SM*) in g·m^−2^, and fabric thickness (*Th*) in mm, respectively. *Rm* for both *M* and *S* indicates the resistance values in mΩ, while *D%* denotes the coefficient of electrical anisotropy and is expressed in %.

**Figure 16:**
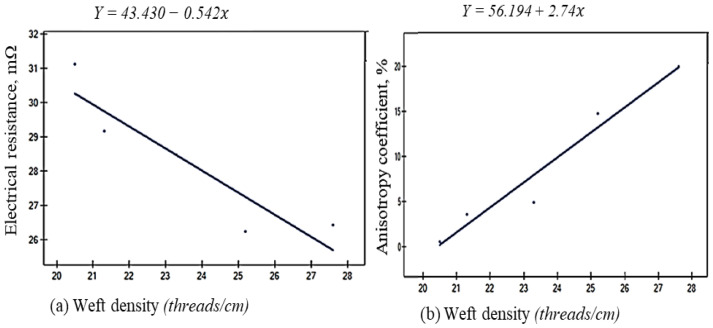
Impact of weft density (*WeD*) on (**a**) electrical resistance (*Rm*) of woven fabrics; (**b**) electrical anisotropy (*D*%) of woven fabrics.

**Figure 17:**
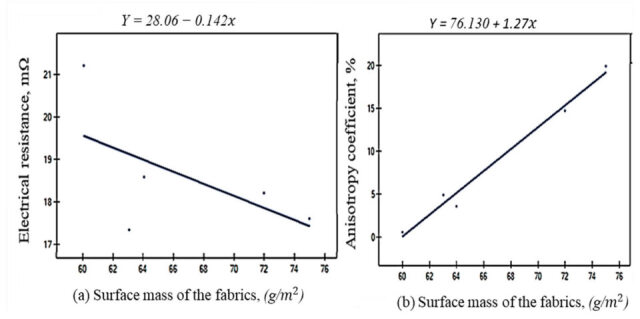
Impact of the surface mass of the fabrics (*SM*) on (**a**) electrical resistance (*Rm*) of woven fabrics; (**b**) electrical anisotropy (*D%*) of woven fabrics.

The *r*-Pearson correlation matrix (Table 7) examines the relationships between key parameters, including weft density (*WeD*), surface mass (*SM*), thickness (*Th*), electrical anisotropy coefficient (*D%*), and electrical resistance (*Rm*). The analysis reveals that weft density (*WeD*) is a key parameter influencing changes in electrical resistance and anisotropy (*D%*). However, in the case of *Rm S*, the magnitude of electrical resistance is less affected by variations in physical parameters. The warp density (*WaD*) does not significantly influence electrical resistance in the determined electrode configuration (*M*-*S*). In contrast, surface mass (*SM*) and weft density (*WeD*) are positively related to electrical anisotropy (*D*%) in both independent electrode arrangements (*M* and *S*). Notably, *WeD*, *SM*, *Th*, *D%M*, and *D%S* exhibit strong positive correlations (*r* > 0.75), indicating a high degree of interdependence. The governing factor of weft densities influenced other parameters, such as surface mass and fabric thickness, which in turn affected the anisotropic properties of electrical resistance. On the other hand, *Rm M* shows strong negative correlations with *WeD*, *SM*, *Th*, *D%M*, and *D%S* (*r* < −0.75), highlighting a significant inverse relationship. These findings provide insights into the influence of *WeD*, *SM*, and *Th* on electrical resistance across the two electrode arrangements (*M* and *S*). The linear regression analysis between key parameters and their impacts on *Rm* and *D%* is illustrated in Figure 16 and Figure 17, demonstrating how well the data points fit the linear model and indicating the proportion of the variation in the physical parameters explained by the independent variables in Table 8.

The values of the linear relationship *r*^2^ indicate that the linear model effectively explains both the electrical resistance and its variability based on key parameters (*WeD*, *SM*, and *Th*). All variables exhibit strong linear relationships (*r*^2^ > 0.84), with higher values (closer to 1) suggesting a better fit. The best fits are observed for surface mass and fabric thickness. Notably, fabric thickness shows a strong correlation with electrical anisotropy *D%.* Fabric thickness shows the strongest correlation with electrical anisotropy under the *S* electrode arrangement (*r*^2^ = 0.9993). This is linked to the production of fabrics with manageable surface irregularities, which play a key role in directional resistance, even within a reduced area of the fabrics limited by four electrodes. The governing factor that greatly affected the electrical resistance was the surface mass of the woven fabrics, i.e., *r*^2^ = 0.8713, followed by the weft density of the woven samples. All *p*-values were statistically significant (*p*-value < 0.05), confirming the validity of the observed linear relationships. The most decisive significance is observed for fabric thickness with the anisotropy coefficient, which is the significant level (*p*-value = 0.000). The linear relationship and the directions between the electrical properties (*Rm* and *D%*) and the independent fabric parameters are presented in Figure 16 and Figure 17.

In the linear relationship analysis between the electrical resistance and weft density shown in Figure 16a, higher weft density results in a more compact fabric structure with increased conductive paths on the surface and fewer air gaps between yarns. The electrical resistance decreases as the number of conductive yarns increases from 20.5 threads/cm to 27.6 threads/cm due to the enhanced conductive pathways provided by the additional silver-plated material (yarns), facilitating more efficient current flow throughout the fabric. The observed changes are mainly due to variations in weft density, while warp density remains nearly constant, as shown in Table 1. Increasing the weft density creates structural differences between the warp and weft yarns, which alter the fabric’s electrical properties. Linear regression analysis shows that higher weft density is associated with an increase in the anisotropy coefficient (*D%*), highlighting the fabric’s anisotropic structure. This is because the availability of higher weft threads, without a corresponding rise in warp threads, increases the directional dependence of electrical resistance, as shown in Figure 16b. In denser regions, the conductive area expands, and the resistive path length shortens, resulting in lower electrical resistance. This is due to the increased contact points and conductive pathways, allowing for smoother current flow. Similar to the effect of weft density, the surface mass (*SM*) of the fabric significantly influences the electrical parameters *Rm* and *D%* (Figure 17a,b). An increase in *SM* enhances electrical conductivity by providing more pathways for charge transport. Moreover, higher *SM* leads to an increase in the anisotropy coefficient (*D%*) due to the preferential alignment of conductive paths along the fabric’s length. This alignment lowers resistance along the lengthwise direction compared to the perpendicular (width) direction, inducing directional dependence in the fabric’s electrical properties.

The increase in anisotropy is a consequence of uneven fiber orientation and material distribution across the surface. Fabric thickness also plays a significant role in influencing electrical resistance, as it is closely related to surface variability and irregularities. The sample W27 is thicker (0.29 mm) than W19 (0.22 mm); it exhibits lower surface roughness (as indicated by lower *sRa* and s*Rq* values) and a reduced electrical resistance (26.422 mΩ compared to 31.114 mΩ for W19). This apparent contradiction highlights that fabric thickness alone does not dictate surface roughness or electrical resistance. Instead, microstructural factors—such as weft yarn density, the degree of yarn alignment, and the continuity of conductive paths—play a more dominant role. In the case of W27, the higher weft density leads to an increased number of conductive elements per unit area and improved yarn alignment, contributing to both a smoother surface and enhanced conductivity. W27 incorporates more conductive yarns than W19, which enhances the formation of continuous electrical pathways and thus reduces resistance. Therefore, this study’s observed trends are specific to the tested configurations and yarns. It is important to emphasize that these outcomes may vary depending on the type of yarn, the coating material, and the weave structure. Further investigation across different fabric systems is required to generalize these findings.

In this study, the fabrics constructed with silver-plated polyamide yarns, such as sample W27, were observed to have greater surface mass and thickness than sample W19; however, W27 exhibits lower surface roughness (s*Ra* and s*Rq*) and reduced electrical resistance. This suggests that, within this specific textile material, microstructural characteristics—particularly weft yarn density (Figure 16a)—play a more decisive role in determining electrical performance. The higher weft density in W27 enhances the fiber integrity and continuity of conductive pathways, resulting in a smoother surface and improved conductivity. While prior studies have established that yarn density and alignment influence conductive textile performance [45,52], the results in this study reveal material-specific thresholds and surface profile mechanisms. In comparing samples W19 and W27, a notable reduction in surface roughness (*Ra*) was observed, decreasing from 0.051 mm in W19 to 0.027 mm in W27 (Table 1), representing a 47% decrease. Correspondingly, the mean electrical resistance (*Rm*) decreased from 31.11 Ω in W19 to 26.42 Ω in W27 (Table 4), indicating a 15% reduction. Additionally, the anisotropy coefficient (*D%*) increased significantly from 0.6% in W19 to 29.9% in W27. These findings suggest that the smoother surface topology in W27, as evidenced by the lower *Ra* value, contributes to enhanced electrical conductivity, likely due to improved contact between conductive elements and reduced electron scattering. The substantial increase in *D%* indicates a higher degree of directional dependence in electrical resistance, which may be attributed to the specific alignment and distribution of conductive yarns in W27. This anisotropy underscores the importance of microstructural characteristics, such as yarn orientation and density, in influencing the electrical properties of conductive textiles. The angular distribution of material orientations—categorized explicitly into three primary directions: 0°/180° (warp) straight, 90°/270° (weft), and 45°/135°/225°/315° (bias)—significantly influences the electrical resistance (*Rm*) and anisotropy coefficient (*D%*) of woven conductive fabrics. The measurements, detailed in Table 2 and Table 3, reveal that resistance values vary with the direction of current flow relative to the fabric’s structural orientation. Notably, samples exhibit lower resistance along the weft direction (0°/180°) compared to the weft and bias directions, indicating anisotropic conductive behavior. These findings are consistent with previous studies that have reported directional dependence of electrical resistance in textile materials. The observed variations underscore the importance of considering fabric orientation in the design and application of electro-conductive textiles, particularly for devices where directional conductivity is critical.

Overall, the comparative analysis between W19 and W27 highlights the critical role of surface morphology and internal fabric structure in determining the electrical performance of conductive textiles. These insights emphasize the need for precise control over fabric construction parameters to optimize conductivity for specific applications. It is important to note that these observations are specific to the silver-plated polyamide yarns utilized in this study. Other conductive textile technologies, such as surface-coated fabrics or those incorporating blended conductive fibers, may exhibit different relationships between structural parameters and electrical resistance due to their distinct material compositions and fabrication methods. Therefore, for each conductive textile technology, it is essential to conduct comprehensive evaluations using appropriate methodologies to accurately assess their electrical properties. These findings underscore the importance of focusing on internal architectural features to accurately assess and optimize the electrical properties of woven conductive fabrics, especially for sensitive electronic textile applications.

Although the detailed behavior of electric current penetration in textile materials warrants further investigation using advanced methods such as the Van der Pauw (VdP) technique, it is generally recognized that fabrics with higher surface mass enhance the free path for the flow of electric currents in the fabric structures. In anisotropic woven structures, variations in surface mass often correlate with microstructural inconsistencies, such as differential fiber integration and contact quality between conductive yarns, which are directly linked to the surface roughness parameters (e.g., *sRa, sRq,* and *sRz*25) presented in Table 2. The peaks and valleys quantified by these roughness metrics reflect irregularity of vertical compactness and uneven topography, both of which modulate electrical resistance by increasing the effective path length for current flow. In the den’s fabrics, this irregularity contributes to current scattering and deviation from ideal conductive paths, thus enhancing the anisotropic behavior. Additionally, in the loose fabrics, increased air entrapment between yarns—common in samples that are rougher and have less mass, such as W19 to W23—introduces insulating zones that further disrupt conductivity. Given the structural anisotropy and inherent heterogeneity of textile materials, conventional methods such as the VdP technique alone are insufficient to characterize electrical behavior fully. To address these limitations, this study adopted an integrated methodological framework combining the Van der Pauw technique, four-point probe electrical measurements, and three-dimensional surface profilometry to comprehensively characterize the electrical and topographical properties of the conductive fabrics.

This hybrid framework enabled a more robust analysis of directional conductivity and facilitated improved interpretation of complex current distributions in textiles with non-uniform geometries.

The angular distribution of surface features plays a key role in determining the directional dependency of electrical resistance, as shown in Table 3. Samples with a more uniform angular distribution, such as W19 and W21, display lower anisotropy (*D%*). In contrast, samples with irregular distributions, like W25 and W27, exhibit higher anisotropy. Additionally, the anisotropy coefficient (*D*%) tends to increase with fabric thickness. This is because thicker fabrics have greater directional variations in yarn distribution and structural compactness along the warp and weft directions. The findings from the analysis of electrical resistance, anisotropic properties, and surface characteristics provide a comprehensive understanding of how fabric structure and surface morphology influence electrical behavior. These results highlight the critical role of surface roughness, yarn orientation, and fabric density in determining the ease of current flow and the directional dependency of electrical resistance. In terms of usability, the observed reduction in electrical resistance could significantly impact applications where electrical performance is critical, such as flexible electronics, sensors, and other conductive textile applications. Although the reduction is relatively modest (13–15%), it may still lead to improved overall performance, particularly in terms of enhanced signal transmission and reduced power loss, which are valuable in high-performance environments. Moreover, the anisotropic properties observed in W27, while contributing to increased variability in electrical resistance, may influence its suitability in applications requiring highly uniform resistance. For example, in medical applications such as heart rate or respiratory monitoring, precise and reliable data transmission is essential. In such cases, the increased resistance variability in W27 could limit its effectiveness, and W19 may be a more appropriate choice due to its stable and isotropic electrical properties, which provide greater consistency in performance. From a cost–benefit perspective, the fabrication process for W27 involves additional costs. These include increased material usage (e.g., more weft threads), higher energy consumption, and longer production times associated with tighter fabrics. As a result, W27 could be more expensive compared to W19. For applications where extreme precision or low electrical resistance is not crucial, the 13–15% reduction in resistance may not justify the additional cost, especially in general textile applications where electrical properties are less critical. Based on these findings, the subsequent discussion examines how these factors influence the anisotropic behavior and overall performance of conductive woven fabrics. By examining the interplay between structural parameters and electrical properties, this study offers a foundation for optimizing fabric design for advanced applications in smart textiles and wearable electronics.

## 5. Conclusions

This study investigated how surface roughness and structural parameters, particularly weft density and fabric thickness, influence the electrical resistance and anisotropy of conductive woven fabrics made from silver-plated polyamide yarns, addressing key gaps in optimizing textiles for wearable electronics. Using contactless profilometry (MicroSpy Profile), surface roughness was quantified through parameters such as *sRa* and *sRz*25. Angular distribution analysis revealed that the orientation of surface features plays a critical role in electrical anisotropy.

Fabrics with diagonal surface textures (e.g., W19, W21, and W23) exhibited higher electrical resistance and lower directional dependence. In contrast, those with perpendicular surface orientations (e.g., W25 and W27) demonstrated more uneven current distribution across the fabric surface. These angular distributions generally trended toward diagonal orientations. Electrical resistance (*Rm*) and anisotropic behavior (*D%*) were measured using the Van der Pauw method in conjunction with a four-point probe setup. These measurements were conducted across five fabric variants (W19–W27), with differing weft densities and thicknesses, enabling the analysis of *D*% and *Rm* while considering the fabrics’ anisotropic properties and surface irregularities.

The electrode configuration, ranging from 0° to 315°, enabled the characterization of variability in electrical resistance across multiple surfaces of the fabrics. The electrode arrangement *S-M* enabled the analysis of the electrical properties of the fabric as the fabric area was altered under four electrodes and expanded. This contributed to the study of electrical properties based on a newly developed anisotropic identification system, which compares the relative resistance of isotropic and anisotropic woven fabrics. The results revealed a 13–15% reduction in *Rm* with increased weft density (from W19 to W27), while the anisotropy coefficient (*D*%) increased substantially, from 0.6% to 29.9%. This shows that the amount of material, particularly weft density and surface mass, significantly influences electrical resistance and anisotropic behavior. Higher material content over a specific area improves yarn contact and compactness, facilitating better current flow. As fabric density increases, electrical resistance decreases, and directional conductivity is enhanced. These findings highlight the importance of material quantity in optimizing the electrical performance of conductive textiles. It should be noted that this conclusion is based on woven fabrics made from silver-plated polyamide yarns (Shieldex^®^ 117/17 HCB). Additionally, smoother surface textures (*sRa* decreasing from 0.051 mm to 0.027 mm) strongly correlated with lower electrical resistance (*r* = −0.98, *p*-value < 0.05). This inverse relationship supports the conclusion that tighter yarn packing reduces air gaps and improves electrical conductivity. However, this improvement in conductivity is accompanied by increased anisotropy, suggesting that higher weft density creates directional current pathways, thereby reducing uniformity.

From an application perspective, fabrics with balanced weft–warp designs (e.g., W19), which exhibit less directional dependency, show promising properties for applications where data reliability is highly critical—such as in medical sensors for heart rate monitoring, respiratory monitoring, and other diagnostic technologies—due to their favorable surface conductivity and roughness properties. In contrast, moderate- to high-density fabrics (e.g., W25 and W27), which exhibit higher electrical performance, are more suitable for applications that require fast response times and directional current flow, such as display-integrated fashion, sports monitoring, footwear electronics, and similar wearable devices. This study focused on plain-weave structures. Future research should explore alternative weave types, such as twill or satin, examine the impact of hybrid yarns, and assess how dynamic deformation or bending affects electrical resistance and anisotropy in conductive fabrics.

## Figures and Tables

**Table 1:** Geometrical parameters of produced conductive woven fabrics.

Weave	Sample Code	*WaD*, Threads/cm	*WeD*, Threads/cm	*SM*, g·m^−2^ ± SD ^1^	*Th*, mm ± SD ^1^
Plain (1/1)	W19	20.6	20.5	60 ± 0.001	0.220 ± 0.001
	W21	20.2	21.3	64 ± 0.001	0.225 ± 0.003
	W23	20.5	23.3	63 ± 0.001	0.230 ± 0.004
	W25	20.7	25.2	72 ± 0.001	0.258 ± 0.001
	W27	20.6	27.6	75 ± 0.001	0.290 ± 0.011

^1^ SD—the standard deviation.

**Table 2:** The electrical property and roughness profile values of woven fabrics.

Samples	W19	W21	W23	W25	W27
*sRa*, mm	0.051	0.047	0.042	0.030	0.027
*sRq*, mm	0.087	0.079	0.072	0.056	0.054
*sRz*25, mm	0.659	0.570	0.547	0.534	0.548
*sRsk*	−2.657	−2.539	−2.652	−3.549	−3.482
*sRmax*25, mm	1.213	0.914	0.939	0.974	0.921
*sRku*	12.454	10.007	11.669	20.011	21.090
*Rm M*, mΩ	31.114	29.154	28.475	26.238	26.422
*Rm S*, mΩ	21.211	18.579	17.334	18.208	17.604
*D%M*, %	0.6	3.6	4.9	14.7	19.9
*D%S*, %	0.2	1.2	2.0	7.2	13.7

**Table 3:** Overall comparison of angle distributions with the relative prominence.

Quadrant	W19 (*θ*,*ps*)	W21 (*θ*,*ps*)	W23 (*θ*,*ps*)	W25 (*θ*,*ps*)	W27 (*θ*,*ps*)	Observations
1st	45°, 0.44%	44°, 0.46%	44°, 0.40%	0°, 0.37%	89°, 0.37%	W21 > W19 > W21 > W25 > (*ps*)
2nd	133°, 0.42%	135°, 0.43%	134°, 0.41%	178°, 0.38%	108°, 0.37%	W21 > W19 ≈ W23 > W25 > W27 (*ps*)
3rd	226°, 0.43%	225°, 0.43%	224°, 0.41%	183°, 0.37%	269°, 0.35%	W19 = W21 > W23 > W25 > W27 (*ps*)
4th	314°, 0.41%	313°, 0.44%	312°, 0.40%	315°, 0.39%	272°, 0.36%	W21 > W19 ≈ W21 > W25 > W27 (*ps*)

**Table 4:** Electrical resistance and anisotropic properties.

	Sample	Electrode Arrangement	*Rm*, mΩ	Normalized *Rm*	*Diso*, mΩ	*Daniso*, mΩ	*D%*, %
Fabric Variants	W19	*M*	31.11	1.00	190.51	191.67	0.6
		*S*	22.68	1.00	138.79	139.12	0.2
	W21	*M*	29.15	0.60	178.51	184.90	3.6
		*S*	21.19	0.30	129.31	130.88	1.2
	W23	*M*	28.48	0.52	174.35	182.88	4.9
		*S*	20.81	0.20	127.40	129.93	2.0
	W25	*M*	26.24	0.00	160.65	184.26	14.7
		*S*	19.17	0.00	117.37	125.83	7.2
	W27	*M*	26.42	0.03	161.78	210.10	29.9
		*S*	19.67	0.17	120.44	136.89	13.7

**Table 5:** Correlation analysis of electrical resistance and roughness values of plain-weave fabrics.

*r*-Pearson Coefficient	*sRa*	*sRq*	*sRz25*	*sRmax25*	*sRsk*	*sRku*	*Rm M*	*Rm S*	*D%M*	*D%S*
*sRa*		**0.9957**	0.7460	0.5463	**0.9380**	**−0.9248**	**0.9654**	0.6599	**−0.9835**	**−0.9336**
*sRq*	**0.9957**		0.8011	0.6011	**0.9176**	**−0.8927**	**0.9847**	0.7098	**−0.9670**	**−0.9025**
*sRz25*	0.7460	0.8011		**0.9096**	0.5261	−0.4493	**0.8832**	**0.9520**	−0.6631	−0.5512
*sRmax25*	0.5463	0.6011	**0.9096**		0.2630	−0.2020	0.7031	**0.9313**	−0.5097	−0.4462
*sRsk*	**0.9380**	**0.9176**	0.5261	0.2630		**−0.9888**	0.8605	0.3727	**−0.9347**	**−0.8802**
*sRku*	**−0.9248**	**−0.8927**	−0.4493	−0.2020	**−0.9888**		−0.8138	−0.3271	**0.9382**	**0.9129**
*Rm M*	**0.9654**	**0.9847**	**0.8832**	0.7031	0.8605	−0.8138		0.7800	**−0.9227**	−0.8352
*Rm S*	0.6599	0.7098	**0.9520**	**0.9313**	0.3727	−0.3271	0.7800		−0.5879	−0.5221
*D%M*	**−0.9835**	**−0.9670**	−0.6631	−0.5097	**−0.9347**	**0.9382**	**−0.9227**	−0.5879		**0.9787**
*D%S*	**−0.9336**	**−0.9025**	−0.5512	−0.4462	**−0.8802**	**0.9129**	−0.8352	−0.5221	**0.9787**	

**Table 6:** Mann–Whitney U test for resistance comparison of the arrangement of the electrodes, *M*-*S*.

Sample	W19	W21	W23	W25	W27
M-W.U test	*M*-*S*	*M*-*S*	*M*-*S*	*M-S*	*M*-*S*
*p*-value	**0.000078**	**0.000155**	**0.001845**	0.104816	0.104816
*Z* statistic	3.310597	3.308162	2.888078	1.627826	1.627826

**Table 7:** Correlation analysis between physical parameters and electrical resistance variables (*Rm* and *D%*).

			*r*-Pearson Coefficient					
	*WaD*	*WeD*	*SM*	*Th*	*Rm M*	*Rm S*	*D%M*	*D%S*
*WaD*		0.4946	0.4115	0.4713	−0.3672	0.0762	0.4825	0.4578
*WeD*	0.4946		**0.9442**	**0.9673**	**−0.9173**	−0.6854	**0.9752**	**0.9628**
*SM*	0.4115	**0.9442**		**0.9611**	**−0.9334**	−0.5851	**0.9912**	**0.9563**
*Th*	0.4713	**0.9673**	**0.9611**		−0.8461	−0.5279	**0.9832**	**0.9997**
*Rm M*	−0.3672	**−0.9173**	**−0.9334**	−0.8461		**0.7800**	**−0.9227**	−0.8352
*Rm S*	0.0762	−0.6854	−0.5851	−0.5279	**0.7800**		−0.5879	−0.5221
*D%M*	0.4825	**0.9752**	**0.9912**	**0.9832**	**−0.9227**	−0.5879		**0.9787**
*D%S*	0.4578	**0.9628**	**0.9563**	**0.9997**	**−0.8352**	−0.5221	**0.9787**	

**Table 8:** The linear regression of fabric parameters concerning their electrical conductivity.

Analyzed Variables	*WeD*	*WeD*	*WeD*	*SM*	*SM*	*SM*	*Th*	*Th*
	*Rm M*	*D%M*	*D%S*	*Rm M*	*D%M*	*D%S*	*D%M*	*D%S*
Regression (*r*^2^)	0.8415	0.9511	0.9271	0.8713	0.9824	0.9145	0.9667	0.9993
*p*-value	**0.0282**	**0.0047**	**0.0086**	**0.0204**	**0.0010**	**0.0109**	**0.0026**	**0.0000**

## Data Availability

The original contributions presented in this study are included in the article. Further inquiries can be directed to the corresponding author.
